# Recent advances in hearing conservation programmes: A systematic review

**DOI:** 10.4102/sajcd.v67i2.675

**Published:** 2020-03-03

**Authors:** Nomfundo F. Moroe, Katijah Khoza-Shangase

**Affiliations:** 1 Department of Speech Pathology and Audiology, Faculty of Humanities, School of Human and Community Development, University of the Witwatersrand, Johannesburg, South Africa; 2 Department of Speech Pathology and Audiology, Faculty of Humanities, University of the Witwatersrand, Johannesburg, South Africa

**Keywords:** Advances, Hearing conservation programmes, Industry, Innovation, Management, Occupational, Policies, Recent

## Abstract

**Background:**

Current evidence from low- and middle-income (LAMI) countries, such as South Africa, indicates that occupational noise-induced hearing loss (ONIHL) continues to be a health and safety challenge for the mining industry. There is also evidence of hearing conservation programmes (HCPs) being implemented with limited success.

**Objectives:**

The aim of this study was to explore and document current evidence reflecting recent advances in HCPs in order to identify gaps within the South African HCPs.

**Method:**

A systematic literature review was conducted in line with the Preferred Reporting Items for Systematic Reviews and Meta-Analysis. Electronic databases including Sage, Science Direct, PubMed, Scopus MEDLINE, ProQuest and Google Scholar were searched for potential studies published in English between 2010 and 2019 reporting on recent advances in HCPs within the mining industry.

**Results:**

The study findings revealed a number of important recent advances internationally, which require deliberation for possible implementation within the South African HCPs context. These advances have been presented under seven themes: (1) the use of metrics, (2) pharmacological interventions and hair cell regeneration, (3) artificial neural network, (4) audiology assessment measures, (5) noise monitoring advances, (6) conceptual approaches to HCPs and (7) buying quiet.

**Conclusion:**

The study findings raise important advances that may have significant implications for HCPs in LAMI countries where ONIHL remains a highly prevalent occupational health challenge. Establishing feasibility and efficacy of these advances in these contexts to ensure contextual relevance and responsiveness is one of the recommendations to facilitate the success of HCPs targets.

## Introduction

A recent systematic review on the management of occupational noise-induced hearing loss (ONIHL) in the mining sector in Africa between 1994 and 2016 revealed that there is a dearth of research on its management (Moroe, Khoza-Shangase, Kanji, & Ntlhakana, 2018). Findings from this study revealed limited research, often conducted with small sample sizes, limiting the generalisation of findings – research that limited its focus on some aspects of hearing conservation programme (HCP) pillars instead of comprehensive review of these programmes within the African context. These authors argue that the limited research and the nature of the research in this field in Africa is a contributing factor towards the documented failure of HCPs in this context as evidenced by the ONIHL in African countries, which is still on the rise. Consequently, these authors raise an implication for future studies in HCPs to facilitate implementation of successful programmes within the mining sector.

An HCP is required when any person in the workplace is exposed to excessive noise. Excessive noise is defined as an equivalent sound pressure level of 85 decibels (averaged) (dB [A]) or more over an 8-h workday (Workplace Safety and Health Council, 2010). The objectives of an HCP are to protect exposed employees from the adverse effects of noise and to minimise the risks associated with workplace noise exposure, thereby preventing ONIHL (Workplace Safety and Health Council, 2010). Occupational noise-induced hearing loss is an occupational medical condition (Lie, Skogstad, Johnsen, Engdahl, & Tambs, 2015) characterised by a permanent sensorineural hearing loss because of excessive exposure to high levels of noise in the workplace (Nelson, Nelson, Concha-Barrientos, & Fingerhut, 2005; Thorne, 2006). Globally, it is said to be the number one work-related disability and the second most common form of acquired hearing loss (Mostaghaci et al., 2013; Ritzel & McCrary-Quarles, 2008).

While hearing loss is not considered a life-threatening condition (Campo, Morata, & Hong, 2013; Hong, Kerr, Poling, & Dhar, 2013; Le, Straatman, Lea, & Westerberg, 2017), if left unmanaged, it can significantly lead to adverse consequences on the health, safety and economic outlook of the affected individuals, their families, societies and the state as well (Moroe, 2018b). Furthermore, in low- and middle-income (LAMI) countries such as South Africa, the effects of ONIHL should not be underestimated as South Africa is currently faced with a quadruple burden of disease, increased unemployment rates, and political and economic instability (Gray & Vawda, 2016; Leboea, 2017; Lehohla, 2017); hence, there is the importance of successful HCPs.

In South Africa, HCPs were first officially implemented in 1994, following the Leon Commission of Enquiry into Health and Safety. In 2003, the South African Mine Health and Safety Council (MHSC), comprising State, Labour and Employer representatives, in consultation with various mine houses, implemented the MHSC 2003 ONIHL milestones (Phillips, Heyns, & Nelson, 2007; Strauss, Swanepoel, Becker, Eloff, & Hall, 2014) as an improvement to the HCP implemented in 1994. These improved milestones were further refined in 2014 (Moroe, 2018). Moroe (2018) argues that the refinement of HCPs alludes to the complex nature of HCPs within the South African context. Furthermore, these refinements could be driven by the rapid advances in technology over the years, which may have a significant influence on HCPs.

Hearing conservation programmes are complex in that all seven pillars comprising HCPs, which include periodic noise exposure monitoring, engineering controls, administrative controls, personal hearing protection, audiometric evaluations, employee and/or management education, and training and record keeping (Hong et al., 2013; Moroe et al., 2018), must be implemented in order to yield the expected outputs. Inherent to an HCP is the influence of different stakeholders who have a key role in the implementation of these programmes. Furthermore, keeping up with research into advances in technology seems to influence the implementation of HCPs.

In fact, according to Brauch (2017):

We live in an era of unprecedented technological advancement that impacts every aspect of our lives, from the way we shop and travel to the way we communicate with friends and family. These trends are resulting in new methods and tools that change the way safety professionals and industrial hygienists prevent hearing loss. (p. 1)

Brauch (2017) asserts that advances in technology with regard to HCPs are evidenced by the use of smartphones, low-cost sensors and the growing interest by government agencies in promoting and implementing HCPs to ensure worker safety and health outcomes. Brauch (2017) argues that, for instance, ‘buying quiet’ as an engineering control strategy is not always feasible or cost-effective, and traditional noisy machining systems can easily be replaced by more versatile and affordable low noise printers.

Medical advancements are also a consideration for HCPs, particularly in the otoprotective agents, which might greatly assist in preventing the impact of noise on the ear. There is sufficient evidence to show that the development of drugs that prevent or treat hearing loss is an increasing business. This rapid growth has been attributed to advancements in drug delivery to the inner ear (Henderson & Tanaka, 2009). Tieu and Campbell (2013) provide a comprehensive review of pharmacologic otoprotective agents in or approaching clinical trials, and how these elucidate mechanisms of noise-induced hearing loss (NIHL), where both prophylactic and rescue agents are included. These authors reviewed classes of agents including antioxidants, vasodilators and glucocorticoids, and concluded that although no pharmacologic agent is yet approved by the Food and Drug Administration (FDA) for clinical use to prevent or treat NIHL during the time of their review, they expect that this situation would change within a decade or so to allow for at least one of these agents to be available for clinical use – another reason for the importance of the current systematic review study.

There is therefore a need to review evidence on recent advances in HCPs and ONIHL in order to update occupational audiologists and relevant stakeholders for successful target outcomes within this sector – particularly within the LAMI contexts where limited resources and strict healthcare priorities call for primary prevention measures to be successfully implemented. This systematic review and focus on ONIHL is also timely as it aligns well with the World Health Assembly’s (2017) focus, which is deliberately highlighting the prevention of deafness and NIHL, particularly in LAMI countries such as South Africa – as 90% of the population with hearing loss resides in these countries. Within the South African context, as early as 2013, the Minerals Council South Africa, formerly known as the Chamber of Mines of South Africa, called for innovative and comprehensive ways of managing ONIHL in the mining sector (Booyens, 2013) – hence the relevance of the current study. The main objective of the current study is to explore and document recent advances in HCPs through a systematic review methodology.

## Methods

### Data sources and literature search

In identifying studies on the recent advances in HCPs, a systematic literature review was conducted following the Preferred Reporting Items for Systematic Reviews and Meta-Analyses (PRISMA) guidelines. Therefore, electronic bibliographic databases including Science Direct, PubMed, Scopus MEDLINE, ProQuest and Google Scholar were searched for potential studies. The following search terms (PubMed mesh terms) were included: ‘recent’ OR ‘advances’ AND ‘ management’ AND ‘hearing conservation programmes’, OR ‘policies’ OR ‘industry’, ‘occupational’, ‘innovative’.

### Inclusion and exclusion criteria

Articles between the 2010 and 2019 time frame were selected for inclusion in the study if they were original pieces of scientific work or reports published in peer-reviewed scientific journals, with a focus on recent advances on HCPs or the management of ONIHL, and were published in English. Articles that did not meet these inclusion criteria were excluded from the study.

### Data extraction and synthesis

Articles selected for inclusion were independently identified by the researchers (N.F.M. and K.K.-S.). In instances where there were disagreements on papers selected for inclusion, these were resolved through discussion and consensus of both authors. Where consensus was not reached, N.M. as the primary investigator made the final decision. After agreeing on articles to be included, the researchers then proceeded to provide a narrative synthesis of the findings from the studies meeting the inclusion criteria. The synthesis included the study objectives, study design, study setting and reported strategy which is regarded as an advancement for the purposes of this manuscript.

**FIGURE 1:**
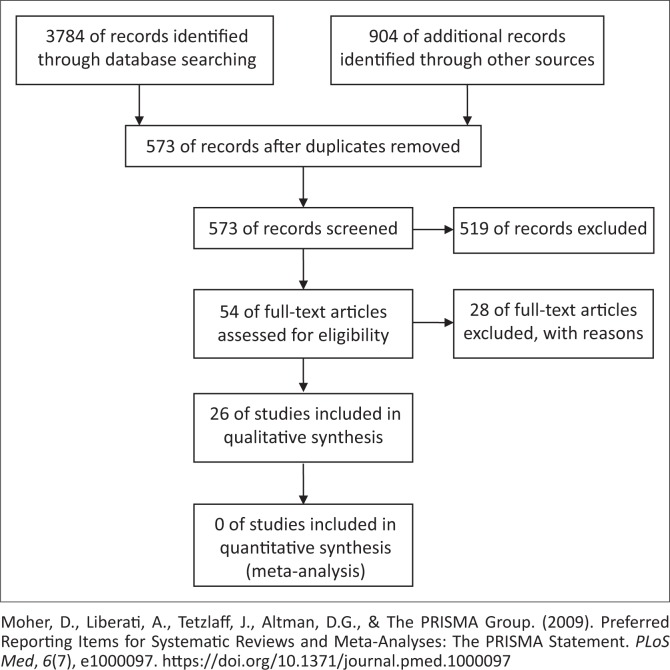
The Preferred Reporting Items for Systematic Reviews and Meta-Analyses flow diagram describing the process of study selection.

A total of 4688 studies were identified for a potential analysis in this study. Of the studies, 3784 records were identified through the aforementioned database search, while the remaining 904 studies were identified through manual searches of references of the identified studies. In the process of collating and organising the studies, 4115 studies were removed as these were duplicates; thus, only 573 studies were considered. Of the 573 remaining studies, 519 were excluded based on the titles and/or abstracts deemed not in line with the focus of the study. Hence, 54 studies were assessed for eligibility and from this 28 were excluded as they did not meet the inclusion criteria of the current study, meaning that these studies were either conducted before the stipulated time frame or the focus was not contributing towards recent advances in the management of ONIHL. Finally, 26 studies were included for analysis in the current study (see Figure 1).

### Ethical considerations

This article followed all ethical standards for a research without direct contact with human or animal subjects.

## Results and discussion

Findings of the current systematic review indicated recent advances towards assessment and management of ONIHL. In total, 26 studies were reviewed, of which 16 were from high-income countries and the remaining 10 were from the LAMI regions (see Table 1). Of the remaining 10, only two were from Africa. Qualitative analysis revealed the emergence of seven themes under which the recent advances can be captured. The fact that glaringly limited research conducted on HCPs in Africa was noted is a significant drawback to the collation of contextually relevant evidence that can be used to implement effective HCPs, as cautioned by Moroe et al. (2018), and the fact that most of this evidence was from South Africa alone is an even bigger concern. These themes include advances in the use of metrics, pharmacological interventions and hair cell regeneration, artificial neural networks (ANNs), audiology assessment measures, noise monitoring advances, conceptual approaches to HCPs, and buying quiet.

**TABLE 1:** Details of study characteristics of each included study.

Authors and date	Research title	Aim	Recent advance	Country	Contribution
Davies et al. (2012)	The use of the kurtosis metric in the evaluation of occupational hearing loss in workers in China: Implications for hearing risk assessment	Examine the value of using the statistical metric, kurtosis [β(t)], along with an energy metric to determine the hazard to hearing from high-level industrial noise environments, and the accuracy of the International Organization for Standardization (ISO-1999:1990) model for median noise-induced permanent threshold shift (NIPTS) estimates with actual recent epidemiological data	Metric	China	The kurtosis metric is an important variable in determining the hazards to hearing posed by a high-level industrial noise environment for hearing conservation purposes. The ISO-1999 predictive model does not accurately estimate the degree of median NIPTS incurred to high-level kurtosis industrial noise.
Moroe et al. (2019)	A proposed preliminary model for monitoring hearing conservation programmes in the mining sector in South Africa	Propose the use of a feedback-based noise monitoring model as a tool for monitoring and managing ONIHL in South Africa’s mining sector		South Africa	This model is a basic static feedback model with practical applications such as estimating, monitoring and providing quantitative information to aid miners, mining administrators and policymakers in decision-making around HCPs. Additionally, the model could form part of an early intervention and management strategy for ONIHL in the workplace. The strength of this model, although currently static, is that it encompasses all the pillars of HCPs and takes into account the policies concerned with the management of ONIHL in the mining sector.
*Al-Dayyeni et al. (2018)*	Investigations of auditory filters-based excitation patterns for assessment of noise-induced hearing loss	Application of two auditory filters, dual-resonance nonlinear (DRNL) filter and rounded-exponential (ROEX) filters to create two excitation potentials (EPs) – the velocity EP and the loudness EP, respectively. Proposed two noise hazard metrics based on two proposed EPs to evaluate hazardous levels caused by different types of noise		United States	Excitation potentials can reflect the responses of the BM to different types of noise. For Gaussian noise there is a frequency shift between the velocity EP and the loudness EP. Both EPs can be used for the assessment of NIHL.
Choi et al. (2014)	Therapeutic effects of orally administrated antioxidant drugs on acute noise-induced hearing loss	Investigate the dose-dependent therapeutic effect of the orally administrated antioxidant drugs (4-hydroxy alpha-phenyl-tert-butylnitrone [4-OHPBN] and N-acetyl-L-cysteine [NAC]) on acute noise-induced hearing loss because oral administration is the most commonly used method of drug administration owing to its convenience, safety and economical efficiency	Pharmacological interventions	Republic of Korea	Orally administered drugs can treat acute noise-induced hearing loss in a dose-dependent manner. This suggests that oral administration was effective in treating acute noise-induced hearing loss as in intraperitoneal administration.
Choi and Choi (2015)	Noise-induced neural degeneration and therapeutic effect of antioxidant drugs	Review structural and functional changes and basic mechanisms induced by noise exposure in the cochlea and the brain and the therapeutic effects of a variety of antioxidant drugs that have been used in our laboratory for the treatment of noise-induced hearing loss		Republic of Korea	These antioxidant drugs were effective in preventing or treating noise-induced hearing loss. In combination with other antioxidants, antioxidant drugs showed a strong synergistic effect. Furthermore, the successful use of antioxidant drugs depends on the optimal timing and the duration of treatment, which are highly related to the time window of free radical formation induced by noise exposure.
Mukherjea et al. (2011)	The design and screening of drugs to prevent acquired sensorineural hearing loss	Provide insights into current and projected future strategies to prevent sensorineural hearing loss from cisplatin chemotherapy, aminoglycoside antibiotic therapy and noise exposure		United States	Novel delivery systems will provide ways to guide these protective agents to the desired target areas in the inner ear and will circumvent problems with therapeutic interference of anti-tumour and antibiotic agents and will minimise undesired side effects.
Oishi and Schacht (2011)	Emerging treatments for noise-induced hearing loss	Describe the epidemiology, pathology and pathophysiology of noise-induced hearing loss in experimental animals and human. The underlying molecular mechanisms of damage are then discussed as a basis for therapeutic approaches to ameliorate the loss of auditory function. Finally, studies in military, industrial and recreational settings are evaluated. Literature was searched through using the terms ‘noise-induced hearing loss’ and ‘noise trauma’.		United States	With the current pace of development, oral drugs to protect against NIHL should be available within the next 5–10 years. Positive results from ongoing trials combined with additional laboratory tests might accelerate the time from the bench to clinical treatment.
Santaolalla et al. (2013)	Inner ear hair cell regeneration: A look from the past to the future	Review research and clinical application of inner near hair cell regeneration		Spain	Taken together, cochlear gene therapy has been successfully used in the treatment of neurosensory hearing loss and other inner ear disorders. The greatest progress will be achieved, in the near future, in the regeneration of hair cells after use of atonal homolog 1 gene delivered by viral vectors and this may become the best clinical treatment method for certain types of hearing loss.
Tieu and Campbell (2013)	Current pharmacologic otoprotective agents in or approaching clinical trials: How they elucidate mechanisms of noise-induced hearing loss	Review the major classes of otoprotective agents for NIHL that have undergone published peer-reviewed clinical trials, or are currently in or approaching FDA-approved clinical trials		United States	Although no pharmacologic agent is yet approved by the FDA for clinical use to prevent or treat noise-induced hearing loss at this time, it is likely that within the next decade and perhaps within the next few years one or more agents will be available for clinical use. Furthermore, it is expected that through an understanding of the underlying mechanisms and noise-induced hearing loss and otoprotection, even more safe and effective pharmacologic otoprotective agents will be developed.
Zheng and Zuo (2017)	Cochlear hair cell regeneration after noise-induced hearing loss: Does regeneration follow development?	Summarise recent transcriptome profile analysis of zebrafish lateral lines and chick utricles where spontaneous HC regeneration occurs after HC damage		United States	It is now conceivable that the development of appropriate drugs will enable effective HC regeneration *in vivo*. The most effective method for drug delivery is local delivery (by intra- or trans-tympanic or intracochlear injection), which is the most commonly used and effective method of drug delivery in patients with middle or inner ear diseases.
Aliabadi et al. (2015)	Prediction of hearing loss among the noise-exposed workers in a steel factory using artificial intelligence approach	Prediction of hearing loss in noisy workplaces is an important aspect of hearing conservation programme. Artificial intelligence, as a new approach, can be used to predict the complex phenomenon such as hearing loss. Using artificial neural networks, this study aimed to present an empirical model for the prediction of the hearing loss threshold amongst noise-exposed workers.	Artificial neural network	Iran	As occupational hearing loss is frequently non-curable, results of accurate prediction can be used by occupational health experts to modify and improve noise exposure conditions.
Aliabadi et al. (2013)	An empirical technique for predicting noise exposure level in the typical embroidery workrooms using artificial neural networks	Present an empirical technique for predicting the noise level in the typical embroidery processes.		Iran	The developed empirical technique can be a helpful tool to analyse the noise pollution in the mentioned process and can enable acoustics and occupational health professionals to apply hearing conservation programmes.
Rehman et al. (2012)	Predicting noise-induced hearing loss and hearing deterioration index in Malaysian industrial workers using gradient descent with adaptive momentum algorithm	Using age, work-duration and maximum and minimum noise exposure as the main factors involved in the hearing loss. Gradient descent with adaptive momentum (GDAM) algorithm is proposed to predict the NIHL in workers		Malaysia	Hearing Deterioration Index (HDI) is found to be quite high for different sound pressure levels, such as maximum exposure (dB) and average exposure (dB), but is reported normal for minimum exposure
Sabanci et al. (2017)	Noise source determination by using artificial neural network in a metal workshop	Measurement of noises in the machinery laboratory of Karamanoglu Mehmetbey University Technical Sciences Vocational School		Turkey	With this data set, various types of artificial neural networks have been trained by using different training algorithms. By changing the number of neurons in the hidden layer and hidden layer activation functions, it has been tried to obtain the best structure. With the best structure obtained in the study, the minimum errors in MAE and RMSE were determined as 0.018552 and 0.11744, respectively
Badri (2010)	Development of neural networks for noise reduction	Describe the development of neural network models for noise reduction. The networks used to enhance the performance of modelling captured signals by reducing the effect of noise		Jordan	Both analytically and experimentally it has been demonstrated that the additive noise improves the network generalisation on the tested patterns and the training trajectory.
Bakay et al. (2018)	Hidden hearing loss selectively impairs neural adaptation to loud sound environments	Investigate the effect of noise-induced HHL on the ability of neurons in the inferior colliculus (IC) to adapt their responses to repeated switches between relatively quiet and relatively loud sound environments	Audiology assessment measures	United Kingdom	Although noise exposure only impairs threshold adaptation directly, the preserved function of gain adaptation surprisingly aggravates coding deficits for loud environments. These deficits might help to understand why many individuals with seemingly normal hearing struggle to follow a conversation in background noise.
Lobarinas et al. (2017)	Evidence of ‘hidden hearing loss’ following noise exposures that produce robust TTS and ABR wave-I amplitude reductions	Use of a modified startle inhibition paradigm to evaluate whether noise exposures that produce robust TTS and ABR wave-I reduction but not permanent threshold shift (PTS) reduced hearing-in-noise performance.		United States	Hearing-in-noise performance was negatively affected by the noise exposure. However, the effect was observed only at the poorest signal-to-noise ratio and was frequency specific. Although TTS >30 dB 24-h post-noise was a predictor of functional deficits, there was no relationship between the degree of ABR wave-I reduction and the degree of functional impairment.
Plack et al. (2014)	Perceptual consequences of ‘hidden’ hearing loss	Present evidence that a history of noise exposure is associated with difficulties in speech discrimination and temporal processing, even in the absence of any audiometric loss		United Kingdom	Evidence from human temporal bone studies and auditory brainstem response measures suggests that this form of hidden loss is common in humans and may have perceptual consequences regarding the coding of the temporal aspects of sounds. Hidden hearing loss is potentially a major health issue, and investigations are ongoing to identify the causes and consequences of this troubling condition.
Plack et al. (2016)	Toward a diagnostic test for hidden hearing loss	Argument that diagnosis of the condition in individual humans is problematic because of test reliability and lack of a gold standard validation measure		United Kingdom	Despite the obstacles, a diagnostic test for hidden hearing loss is a worthwhile goal, with important implications for clinical practice and health surveillance.
McTague et al. (2013)	Impact of daily noise exposure monitoring on occupational noise exposures in manufacturing workers	Report on an intervention employing the voluntary use of this technology in a worksite setting	Noise monitoring advances	United States	Initial results from this longitudinal study indicate that volunteers find daily noise exposure monitoring to be feasible, and that workers who monitor daily can reduce exposures. The results of subject adherence shed light on the challenges and possibilities of worksite interventions for health and safety.
Michael et al. (2011)	Role of continuous monitoring in a hearing conservation program	Provide evidence that while hearing protective devices (HPDs) can be effective in reducing exposure by 30 dB or more, the protection afforded to an individual is highly variable depending on many factors		United States	For the first time, the occupational hearing conservationist is provided with a quantitative assessment of personal exposure that accounts for the effectiveness of hearing protection across the entire work shift. Besides empowering the employee to better protect himself or herself, these data allow the safety officers to monitor noise exposures daily and intervene after occasional overexposures. With proper intervention and oversight of the programme, a continuous monitoring programme can absolutely prevent occupational hearing loss.
Rabinowitz et al. (2011)	Effect of daily noise exposure monitoring on annual rates of hearing loss in industrial workers	Report on an analysis of the hearing loss experience of a cohort of industrial workers who are enrolled in a mandatory programme to perform daily noise exposure monitoring inside their hearing protection devices in order to determine whether the users of the devices were experiencing less hearing loss than control workers not enrolled in the mandatory programme		United States	Monitoring daily occupational noise exposure inside hearing protection with ongoing administrative feedback apparently reduces the risk of occupational NIHL in industrial workers. Longer follow-up of these workers will help determine the significance of the intervention effect. Intervention studies for the prevention of NIHL need to include appropriate control groups.
Williams et al. (2012)	Usability of a daily noise exposure monitoring device for industrial workers	Trial of a new technology for the prevention of noise-induced hearing loss that allows workers to monitor their noise exposure inside of hearing protection daily; we studied the usability of the daily noise exposure monitoring device.		United States	A novel technology that allows workers to record noise exposures inside of hearing protectors daily has been developed. Current users of the device report positive perception about how the device helps them prevent noise-induced hearing loss. However, in its current version, users reported several usability barriers that are associated with stopping use of the device. These barriers to use should be addressed as the technology progresses.
Bayley et al. (2013)	Wireless headset noise exposure dosimeter	Presentation of systems and methods for measuring noise exposure associated with the use of a wireless headset		United States	
Moroe et al. (2018)	Occupational noise-induced hearing loss in South African large-scale mines: Exploring hearing conservation programmes as complex interventions embedded in a realist approach	Explore whether HCPs are a complex intervention, fitting the predefined criteria for complex interventions	Conceptual approaches to HCPs	South Africa	The success of HCPs in the mining sector depends on conducting contextually evidence-based evaluations, such as realist reviews, which can provide policymakers with contextual evidence for why certain programmes do or do not work in certain settings.
Brereton and Patel (2016)	Buy quiet as a means of reducing workplace	Argument for purchasing quieter machinery as an effective way to avoid risk from occupational exposure to high noise	Buying Quiet	United Kingdom	Buy quiet has the potential to result in lower workplace noise risk if noise information can be made simpler and more reliable. This may require a departure from the harmonised standards approach and a greater reliance on shared information based on noise risk assessed during normal work

ABR, auditory brainstem response; BM, basilar membrane; FDA, Food and Drug Administration; GDAM, gradient descent with adaptive momentum; HCP, hearing conservation programme; HDI, hearing deterioration index; HHL, hidden hearing loss; HPD, hearing protection device; IC, inferior colliculus; MAE, mean absolute error; NIHL, noise-induced hearing loss; ONIHL, occupational noise-induced hearing loss; PTS, permanent threshold shift; RMSE, root mean squared error; TTS, temporary threshold shift.

### The use of metrics

Metrics can be loosely defined as a set of quantitative tools that can be used to assess, monitor, improve or evaluate compliance and success of programmes in order to set and track goals of the implemented programme (Sullivan, McDaniel, McDaniel Limbart, Siegel, & Services, 2004). In this review, three studies by Moroe et al. (2019) and Al-Dayyeni, Sun and Qin (2018) focused on the use of metrics as a recent advancement in HCPs. Davis et al. (2012) examined the value of using the statistical metric, kurtosis, along with an energy metric to determine the hazard to hearing from high-level industrial noise environments, while Moroe et al. (2019) proposed the use of a basic static feedback-based noise monitoring model as a tool for monitoring and managing occupational noise in the workplace. Both these studies used the International Organization for Standardization (ISO-1999:1990) model to estimate and predict the noise-induced permanent threshold shift in people exposed to noise in the workplace. While Davis et al. (2012) used epidemiological data obtained on actual participants, Moroe et al. (2019) used fictitious data; therefore, this model still needs to be validated with actual data. Al-Dayyeni et al. (2018), on the other hand, proposed two noise hazard metrics based on two excitation patterns to evaluate hazardous noise levels caused by different types of noise. In this study, Gaussian noise and single-tone noise are simulated to evaluate performances of the proposed excitation patterns (EPs) and the noise metrics. These metrics were validated on two auditory filters: the dual resonance nonlinear filter and the rounded exponential filter.

The use of metrics as an advancement in the management of ONIHL adds value towards the implementation of HCPs in that, if these models are accurately designed and implemented, they may yield the desired effects of enhancing success of HCPs. Metrics have been included in studies concerned with risk management in occupations with excessive nose exposure (Army Regulation, 2016; Australian Government, 2009; Li, Huang, & Zhang, 2018; Moroe et al., 2019). In these studies, excessive noise exposure is viewed as a risk that needs to be managed. Metrics are versatile in that they can augment any pillar of the HCP. For instance, models can be used in assessing and monitoring the risk of exposure (periodic noise exposure) (Army Regulation, 2016), in measuring and attenuating noise emissions from the machinery (engineering controls) (Nanda, 2012), in improving and evaluating compliance in the implementation of administrative processes and policies designed to mitigate the excessive exposure to the employees (administrative controls), in conducting audiogram baselines and annual checks (audiometric evaluations), in the use of hearing protection devices (personal hearing protection), in how workers are trained on the effects of excessive noise exposure (education and training) as well as in evaluating compliance and success of programmes in order to set and track goals of the implemented programme (monitoring and record keeping) (Moroe et al., 2019). Based on the discussion above, the use of metrics can be considered a valuable advancement in the management of ONIHL. Closely linked to the use of metrics is the use of artificial intelligence in HCPs, another advancement covered in this systematic review.

### Pharmacological interventions and hair cell regeneration

Pharmacological interventions are defined as approaches developed for the prevention or treatment of NIHL using antioxidant drugs to restore the balance between antioxidant defence and the formation of free radicals in the cochlea (Choi & Choi, 2015; Choi, Du, Floyd, & Kopke, 2014). The current review on recent advances in this area shows that there are pharmacological interventions that have a focus on preventing the problem – NIHL – and those that focus on reversing or treating the effects of the problem. As far as the pharmacological interventions that are aimed at preventing the problem are concerned, this systematic review included seven studies (Choi & Choi, 2015; Choi et al., 2014; Mukherjea et al., 2011; Oishi & Schacht, 2011; Santaolalla et al., 2013; Tieu & Campbell, 2013; Zheng & Zuo, 2017).

A study by Choi and Choi (2015) reviewed structural and functional changes and basic mechanisms triggered by noise exposure in the cochlear and the brain as well as the therapeutic effects of an assortment of antioxidant drugs for the treatment of NIHL. This study demonstrated a strong synergistic effect of pharmacological interventions when each antioxidant is used in combination with other antioxidants, depending on optimal timing and the duration of treatment, which is strongly associated with the time window of free radical formation induced by noise exposure. The authors, however, cautioned on the need for further research in this area as the clear relationship between free radical formation and the optimal timing of antioxidant treatment is not yet established. Similarly, Mukherjea et al. (2011) discussed what they term future strategies into the prevention of hearing loss because of excessive noise exposure. According to these authors, novel delivery systems will provide ways to pharmacological interventions to the desired areas in the inner ear, thereby circumventing problems associated with therapeutic interference agents to minimise the side effects.

As far as the pharmacological interventions concerned with reversing or treating the effects of exposure to excessive noise are concerned, three studies were included in this review. Zheng and Zuo (2017) conducted a study to explore the use of drugs to reverse hair cell loss and to promote hair cell regeneration to enable effective hair cells regeneration *in vivo*, while Oishi and Schacht (2011) in a systematic review discussed the pathophysiology of NIHL, the underlying molecular mechanisms of damage and the basis for therapeutic approaches to improve the loss of auditory function. Likewise, Santaolalla et al. (2013) also conducted a literature review and clinical application of inner ear hair cell regeneration. The findings of these authors caution that while drugs can enable effective hair cell regeneration *in vivo*, it is not currently understood how reagents can be delivered effectively to the mature organ of Corti *in vivo* or how drugs pass through the blood-labyrinth barrier in the inner ear fluids – all implications for future studies.

### Artificial neural networks

Artificial neural networks are defined as analytical techniques modelled on the learning processes of human cognitive system and the neurological functions of the brain (Deng, Chen, & Pei, 2008). Deng et al. (2008) state that ANNs work by processing information like biological neurons in the brain and consist of small processing units known as artificial neurons, which can be trained to perform complex calculations. In the current review, five studies (Aliabadi, Farhadian, & Darvishi, 2015; Aliabadi, Golmohammadi, Mansoorizadeh, Khotanlou, & Ohadi Hamadani, 2013; Badri, 2010; Rehman, Nawi, & Ghazali, 2012; Sabanciet et al., 2017) were included on the use of artificial intelligence as a recent advancement in the management of excessive noise exposure in the workplace.

Aliabadi et al. (2013) and Badri (2010) described the development of neural network models for noise reduction, while Aliabadi et al. (2013) additionally presented an empirical technique for predicting the noise levels. Rehman et al. (2012) focused on using age, work duration, and maximum and minimum noise exposure as the main factors involved in the hearing loss. Aliabadi et al. (2015) used artificial intelligence as a new approach to predict hearing loss thresholds amongst noise-exposed workers, while Sabanci et al. (2017) estimated the dominant noise source in a workshop using ANN.

Artificial neural networks are considered as one of the most important artificial intelligence techniques because of their ability to store and apply empirical data (Aliabadi et al., 2013), and they have been successfully applied in fields such as mathematics, engineering, medicine, economics, meteorology, psychology, neurology (Kalogirou, 2006) and now in HCPs. The benefits of ANNs, which can also be successfully applied in designing HCPs, include the following: (1) ANN models may be used as an alternative method in engineering analysis and prediction; (2) they have the ability to handle large and complex systems with many inter-related parameters; (3) they potentially offer better, quicker and more practical predictions than any of the traditional methods; (4) they provide innovative ways of solving design issues instantaneously, with expert opinion on demand; and (5) they are fault tolerant, robust and noise immune (Kalogirou, 2006). With the Fourth Industrial Revolution upon us, with contextual challenges around demand and considering the capacity of audiology and general healthcare manpower in LAMI countries, ANNs seem a valuable recent advance to explore.

### Audiology assessment measures

Bakay, Anderson, Garcia-Lazaro, McAlpine and Schaette (2018), Lobarinas, Spankovich and Le Prell (2017), Plack, Barker and Prendergast (2014) and Plack et al. (2016) have turned to the use of audiological assessment measures as an advanced strategy to diagnose cochlear neuropathology in people exposed to excessive noise. The strength of this strategy lies in its potential to detect early stages of hearing loss in people, who, without further audiological testing, may be mismanaged because of presenting with an audiogram indicating hearing within normal limits. All the above-mentioned authors have, to varying degrees and contexts, explored the relationship amongst noise exposures that produced large temporary threshold shift, significant auditory brain response wave-I reduction and hearing-in-noise deficits in rats. The findings of these studies showed reduced auditory brainstem response (ABR) wave-I amplitudes and a low signal-to-noise ratio in hearing-in-noise performance. Although these studies have only been conducted on rats and there are acknowledged limitations, the strength of the strategy lies in its early detection of cochlear hidden hearing loss in people exposed to hazardous noise.

Literature indicates that ONIHL is gradual (Nandi & Dhatrak, 2008) and it can take about 10–15 years before the full impact of excessive noise is fully realised. By that time, the damage is done and cannot be reversed. In adopting this strategy in HCPs, particularly in audiometric evaluations, monitoring standard threshold shifts, as is the current practice in the South African mining industry (Mine Health and Safety Council, 2016), adding ABR and monitoring wave-I may be beneficial in monitoring the worker’s hearing status before the ‘measurable’ threshold shift occurs, which is the goal of a successful preventive audiology programme.

### Noise monitoring advances

In keeping up with the advances in the use of devices in the management of excessive exposure, researchers such as Michael, Tougaw and Wilkinson (2011), McTague et al. (2013), Williams and Rabinowitz (2012), Michael (2016), Rabinowitz et al. (2011) and Bayley, Woo and Wong (2013) have focused on the noise exposure pillar of the HCP to eliminate noise in the workplace. Bayley et al. (2013) and Michael (2016) in particular have patented devices that can be used for continues noise monitoring in the workplace. Bayley et al. (2013) invented a wireless headset noise exposure dosimeter. This dosimeter has a wireless communications transceiver, a speaker, a non-volatile memory storage, a criterion sound level, threshold sound level, a recorded noise dose measurement, an exchange rate and a processor. Michael (2016), on the other hand, invented a hearing protection device with two microphones to calculate the exposure dosage of periods when the device is worn (primary microphone) and when the device is not worn (secondary microphone). This device provides an accurate measurement of the exposure dosage when worn accurately. McTague et al. (2013) conducted a study where participants were fitted with a noise monitoring device consisting of a dosimeter carried in a pocket or worn on a belt or helmet. This device was connected by wires to a small microphone to record noise exposure under hearing protective devices. Rabinowitz et al. (2011) and Williams and Rabinowitz (2012) used a hearing protection device fitted with a dosimeter attached to a microphone for measuring noise exposures inside the hearing protection device.

The benefits of monitoring noise exposure, if an overexposure is identified, are that the safety representative can intervene appropriately and timely. According to Michael, Tougaw and Wilkinson (2011, p. 197), ‘intervention can take the form of a warning, such as wear the hearing protection devices (HPDs) more effectively tomorrow….’ Additionally, these devices can eliminate the human element in the reporting and thereby minimise reliance on self-correction by the employee.

The use of these gadgets is in line with the sentiments by Brauch (2017) who attested that smart HPDs are capable of providing effective motivators, empowering noise-exposed individuals to take control of their hearing health outcomes. As such, the National Institute for Occupational Safety and Health (NIOSH) has developed alternative fit checking systems that can be used with virtually any type of earplug regardless of the manufacturer (Brauch, 2017). Therefore, the contribution towards effective HPDs in the management of excessive noise exposure in the workplace cannot be underestimated.

### Conceptual approaches to hearing conservation programmes

For the purpose of this systematic review, conceptual approaches are defined as researchers’ synthesis of available evidence used to explain the phenomenon of occupational noise and HCPs, with the goal of mapping out actions required to eliminate excessive exposure to noise in the workplace. In this review, only one study (Moroe, 2018) was included as a recent advance in the management of occupational noise. Moroe (2018) successfully argued for a change in the conceptual framework framing our approach to HCPs where she recommends that HCPs should be viewed as a complex intervention. Complex interventions are defined as interventions built from multiple interacting components, which may act both independently and interdependently (Medical Research Council, 2000; Moore et al., 2015). Complex interventions are generally conducted to improve health, at the individual, organisational, policy or population level, in different fields such as public health research, medical research (Moore et al., 2015) and any public services dealing with complex social interventions such as performance measures, regulations and inspection of funding reforms (Pawson et al., 2005). Seeing HCPs as complex interventions allows for conducting realist reviews, which are concerned with ‘understanding and unpacking the mechanisms by which an intervention works (or fails to work)’ (Rycroft-Malone et al., 2012, p. 1).

Realist approaches focus on theory development while taking into consideration the context when methodically and transparently synthesising results (Pawson, 2006; Pawson et al., 2005; Rycroft-Malone, McCormack, DeCorby, & Hutchinson, 2010). There are four benefits of conducting realist reviews. Firstly, these provide stakeholders and policymakers with enlightenment and empirical evidence on the nature of the programme or intervention implemented in a given setting (Pawson et al., 2005). Secondly, realist reviews assist policymakers to interpret and clearly understand why a programme worked better in one context than in another context, for example, international versus local context. Thirdly, realist reviews provide policymakers with a justification for taking one course of action over another. Lastly, realist reviews alert policymakers about potential problems and specific measures that can be applied to mitigate such problems. Moreover, realist reviews provide explanations rather than judgements around interventions (Pawson et al., 2005). As a recent advance, this conceptual framework ensures holistic and comprehensive, as well as contextually relevant and responsive, implementation of all relevant pillars of HCPs in any given context.

### Buying quiet

Buying quiet is an initiative focused on adopting and promoting the use of quieter machines as a strategy for controlling noise at its source. Brereton and Patel (2016) advocate for designing and manufacturing power tools and equipment as a strategy for reducing the risk of ONIHL. The current authors propose that while buying quiet as a strategy in HCPs is not a recent advance, the suggested partnership between manufacturers and consumers encouraging companies to seek out and demand quieter equipment effectively driving the market to design and create quieter products while demonstrating the cost benefits of doing buying quiet is a recent advance. According to Brereton and Patel (2016), investment in noise controls should in the long run be more common as the market demands quieter products. Exploration of such partnerships will be beneficial to HCPs.

## Conclusion

Hearing conservation programmes are mandated when employees are exposed to excessive noise in the workplace. This review study identified 26 papers that met the predefined inclusion criteria for this systematic review. These papers were further grouped under various themes. The studies that were selected were heterogeneous; therefore, attempts were not made to conduct a quantitative synthesis or meta-analysis; however, the qualitative analysis yielded clear trends indicating advances in seven themed areas, including advances in the use of metrics, pharmacological interventions and hair cell regeneration, artificial intelligence, audiology assessment measures, noise monitoring strategies, conceptual approaches to HCPs and buying quiet. These advances raise important implications for HCPs globally, but particularly so in LAMI countries where ONIHL remains a significant challenge. As evident by the studies included in this review, only two studies were conducted in LAMI countries. This indicates that as far as advances in the management of occupational noise are concerned, local studies have focused on the use of metrics and conceptual approaches. These advances are yet to be carried out practically in order to measure their contribution to recent advances in developing countries. Therefore, there are implications for research aimed at establishing contextually relevant and appropriate HCPs based on the recent advances, which have also been raised by the current findings. Increased efforts for collation of evidence from the African context are needed if Africa is to keep up with recent advances in HCPs. Evidence from these studies may raise implications for training as well as policy formulation within these contexts.
